# Lactation induction in a transgender woman: case report and recommendations for clinical practice

**DOI:** 10.1186/s13006-024-00624-1

**Published:** 2024-03-11

**Authors:** Jojanneke E. van Amesfoort, Norah M. Van Mello, Renate van Genugten

**Affiliations:** 1 Department of Obstetrics and Gynaecology, Amsterdam University Medical Centres, Reproduction and Development Research Institute, Meibergdreef 9, 1105 AZ Amsterdam, The Netherlands; 2 Centre of Expertise On Gender Dysphoria, Amsterdam University Medical Centres, Location VUmc, Amsterdam, the Netherlands; 3 Department of Internal Medicine and Endocrinology, Radboud University, Nijmegen, the Netherlands

**Keywords:** Induced lactation, Transgender, Breastfeeding, Gender dysphoria

## Abstract

**Background:**

We present a case of non-puerperal induced lactation in transgender woman. Medical literature on lactation induction for transgender women is scarce, and the majority of literature and protocols on lactation induction is based on research in cisgender women. Healthcare professionals may lack the precise knowledge about lactation induction and may therefore feel insecure when advice is requested. Subsequently, there is a rising demand for guidelines and support.

**Methods:**

Patient medical record was consulted and a semi-structured interview was conducted to explore the motive for lactation induction, the experience of lactation induction, and to gather additional information about the timeline and course of events.

**Case presentation:**

In this case a 37-year-old transgender woman, who was under the care of the centre of expertise on gender dysphoria in Amsterdam, and in 2020 started lactation induction because she had the wish to breastfeed her future infant. She was in a relationship with a cisgender woman and had been using gender affirming hormone therapy for 13 years. Prior to initiating gender affirming hormone therapy she had cryopreserved her semen. Her partner conceived through Intracytoplasmic Sperm Injection, using our patient’s cryopreserved sperm.

To induce lactation, we implemented a hormone-regimen to mimic pregnancy, using estradiol and progesterone, and a galactogogue; domperidone. Our patient started pumping during treatment. Dosage of progesterone and estradiol were significantly decreased approximately one month before childbirth to mimic delivery and pumping was increased. Our patient started lactating and although the production of milk was low, it was sufficient for supplementary feeding and a positive experience for our patient.

Two weeks after birth, lactation induction was discontinued due to suckling problems of the infant and low milk production.

**Conclusions:**

This case report underlined that lactation induction protocols commonly used for cisgender women are also effective in transgender women. However, the amount of milk produced may not be sufficient for exclusive nursing. Nevertheless, success of induced lactation may be attributed to its importance for parent-infant bonding, rather than the possibility of exclusive chestfeeding.

## Background

Chestfeeding (i.e. gender neutral term for breastfeeding) was, for a long time, solely associated with maternity in cisgender women (i.e. individuals who were assigned female gender at birth and identify as a woman). However, this has increasingly been put into question due to increasing availability and visibility of non-puerperal lactation induction in mothers of adopted children, partners in same-sex relationships and biological mothers of a surrogate pregnancy [1], and puerperal lactation in transgender and gender diverse individuals [2]. Transgender women (i.e. individuals assigned male gender at birth (AMAB) and who affirm their gender as female) and non-binary individuals who are AMAB may wish to chestfeed their infants and constitute a growing population. They may do so through non-puerperal lactation induction [3–5].

Literature and protocols on lactation induction is scarce and merely based on case reports in cisgender women [6–10]. Research focussed on induction of lactation in transgender women is lacking and to our knowledge, only three case reports on lactation induction in transgender women have previously been published [4, 5, 11]. However, transgender women chestfeeding is not limited to these case reports. On mainstream media, cases and experiences of these women are broadcast and may inspire other transgender women to desire this themselves [12–14]. Therefore, the true incidence of lactation induction in transgender women is unknown.

Protocols for induced lactation in transgender women are not readily available. In 2020 Trautner et al. published a cross sectional survey that showed the interest in lactation induction for both providers and patients [15]. The survey was held for attendees of the World Professional Association for Transgender Health (WPATH) in 2018 and a total of 82 attendees responded to the survey (10.5% response rate) [15]. A third of survey respondents expressed that they had met transgender women that disclosed interest in lactation induction. Nevertheless, only a mere 9% of respondents had helped transgender women to induce lactation [15]. The majority of respondents underlined the need for specialized protocols and additional training in lactation induction for transgender women [15].

Chestfeeding offers advantages when compared to infant formula feeding. For instance, chestfed infants have immunological advantages compared to formula fed infants as human milk contains anti-inflammatory agents, secretory IgA and other immunomodulators [16, 17]. Furthermore, chestfeeding offers a lower risk of infants developing certain infections, childhood obesity, asthma, necrotizing enterocolitis and sudden infant death syndrome when compared to infants that are formula-fed [16–21].

It has also been noted that chestfeeding may benefit parent–child attachment [22, 23].

Chestfeeding may provide surrogate mothers emotional benefits, regardless of the amount of milk produced [24]. One could speculate that chestfeeding, as by society it is perceived as feminine, may be gender-affirming for transgender women. Nevertheless, the chances of success and risks of the high levels of hormones in people AMAB remain unknown, and lactation induction not succeeding may be experienced as a disappointment.

Since medical literature is scarce, healthcare professionals may lack the precise knowledge about lactation induction and may therefore feel insecure when advice is requested. Subsequently, there is a rising demand for guidelines and support. Here we report a case of a transgender woman who successfully induced lactation, including a report of her experiences doing so. Furthermore, we will provide recommendations for clinical practice.

## Methods

Written informed consent for this case report was obtained prior to initiation. After receiving written informed consent, medical information for the case presentation was retrieved from the electronic medical record.

For this case report we also conducted a semi-structured interview with the patient to explore the motive for lactation induction, the experience of lactation induction, and to gather additional information about the timeline and course of events. This interview was recorded and transcribed verbatim by the primary author. Information in the interview was analysed and included in the case presentation.

Ethical approval for this study was obtained from the institutional review board at the Amsterdam University Medical Centre. Ethical approval number: 2023.0229.

## Case presentation

### Case history

In January of 2020 a 37-year-old transgender woman (she/her), visited the centre of expertise on gender dysphoria in Amsterdam. She and her partner, a cisgender woman, wanted to fulfil their wish for a child, and both had the desire to chestfeed the infant. They had read about the possibility of lactation induction in transgender women on social media. The main motivation for lactation induction was to enhance parent–child bonding. Furthermore, they were planning to alternate chestfeeding to limit the burden, because the partner was experiencing some physical limitations.

The patient’s medical history was notable for gender incongruence. Hence, she was using gender affirming hormone therapy since October 2007 and had undergone a vaginoplasty in 2010, which was revised in 2011 and 2012. Her surgical history was also significant for an appendectomy because of appendicitis in 2010. The patient’s history was negative for smoking, alcohol and drugs.

Prior to initiating gender affirming hormone therapy she had cryopreserved her semen.

At the time of her visit to the clinic she used estradiol gel 75ug per day, once daily. Her laboratory results from her initial evaluation are displayed in Table 1.

**Table 1:** Laboratory results over time

	***Initial evaluation***	***11 June 2020***	***10 August 2020***	***27 August 2020***
Estradiol	1.1 nmol/L (186 pg/mL)	0.43 nmol/L (73 pg/mL)	0.96 nmol/L (162 pg/mL)	0.52 nmol/L (88 pg/mL)
Progesterone	4.2 nmol/L (1.32 ng/mL)	9.6 nmol/L (3.02 ng/mL)		10.4 nmol/L (3.27 ng/mL)
Testosterone	2.58 nmol/L (74.4 ng/dL)	2.64 nmol/L (76.1 ng/dL)	0.6 nmol/L (0.17 ng/dL)	
Prolactin	0.31 IU/L (14.6 ng/mL)	0.26 nmol/L (12.2 ng/mL)	2.4 IU/L (112.8 ng/mL)	2.09 nmol/L (98.2 ng/mL)
ASAT (SGOT)	41 U/L			
ALAT (SGPT)	48U/L			
Creatinine	68umol/L (0.77 mg/dL)			
Cholesterol	3.8 mmol/L (146.95 mg/dL)			

### Course

As healthcare practitioners of the centre of expertise on gender dysphoria in Amsterdam did not have any experience with lactation induction in transgender women, her request was discussed, and medical research and literature was consulted. At that time only one previous case report, by Reisman and Goldstein, was available of lactation induction in a similar situation and the team decided to apply the same protocol [4]. Low milk production, and thus the possible inability of exclusive nursing were discussed with the patient. Hence, by shared decision making, our patient chose non-puerperal lactation induction.

Her partner conceived after Intracytoplasmic Sperm Injection (ICSI) with cryopreserved sperm of our patient. On 1st June 2020, when her partner was approximately 14 weeks gestation, our patient started non-puerperal lactation induction. The first step was the use of a 150ug estradiol adhesive dermal patch once daily and 100 mg progesterone once daily orally. Laboratory follow up after one month, on the 11th June, are shown in Table 1.

On 22nd June 2020 two pumps estradiol dermal spray (equals approximately 100ug estradiol adhesive patch) was added to the estradiol treatment regime. Our patient started manual stimulation of the nipples and breast.

On the 13th July she started domperidone 10 mg four times daily orally.

On the 20th July domperidone dosage was doubled to 20 mg four times daily orally.

On the 27th July the progesterone dosage was doubled to 100 mg twice daily orally.

She experienced gastrointestinal discomfort due to the use of domperidone, which decreased over the course of time.

Our patient and her partner visited a lactation consultant for lactation support. The lactation consultant was primarily focused on education about massaging of the breasts, pumping techniques and routines. The lactation consultant did not have prior experience with lactation induction in transgender women.

On 10th August 2020, the patient expressed the first drops of fluid from her nipples. Laboratory results are shown in Table 1. Since serum estradiol levels were high, estradiol treatment was decreased by 50ug, to a total of 200ug estradiol daily. Progesterone was increased to three times daily. Laboratory results two weeks later (27th August) are displayed in Table 1. On October 8th 2020, estradiol dosage was decreased by stopping the transdermal spray to a daily dosage of 100ug estradiol daily. On the 16th October progesterone was discontinued and the patient started pumping every four hours, including throughout the night. Every time she pumped a few droplets of milk were released. She produced approximately 2 ml milk per day. Estradiol treatment was decreased to 50ug daily.

On the 5th November 2020 her partner gave birth to their child at 38 weeks and 2 days gestation. Her partner was induced because of gestational diabetes. Both mothers continued or commenced pumping. Our patient produced a maximum of 7 mls a day, which was enough for supplementary feeding. Since the infant had a short lingual frenulum, latching to the nipple was suboptimal and caused suckling problems. She decided to discontinue lactation induction two weeks after the birth of her child, because she experienced the frequent pumping to be exhausting and milk production to be low. Two weeks after the birth her estradiol dosage was increased to 100ug.

**Fig. 1:**
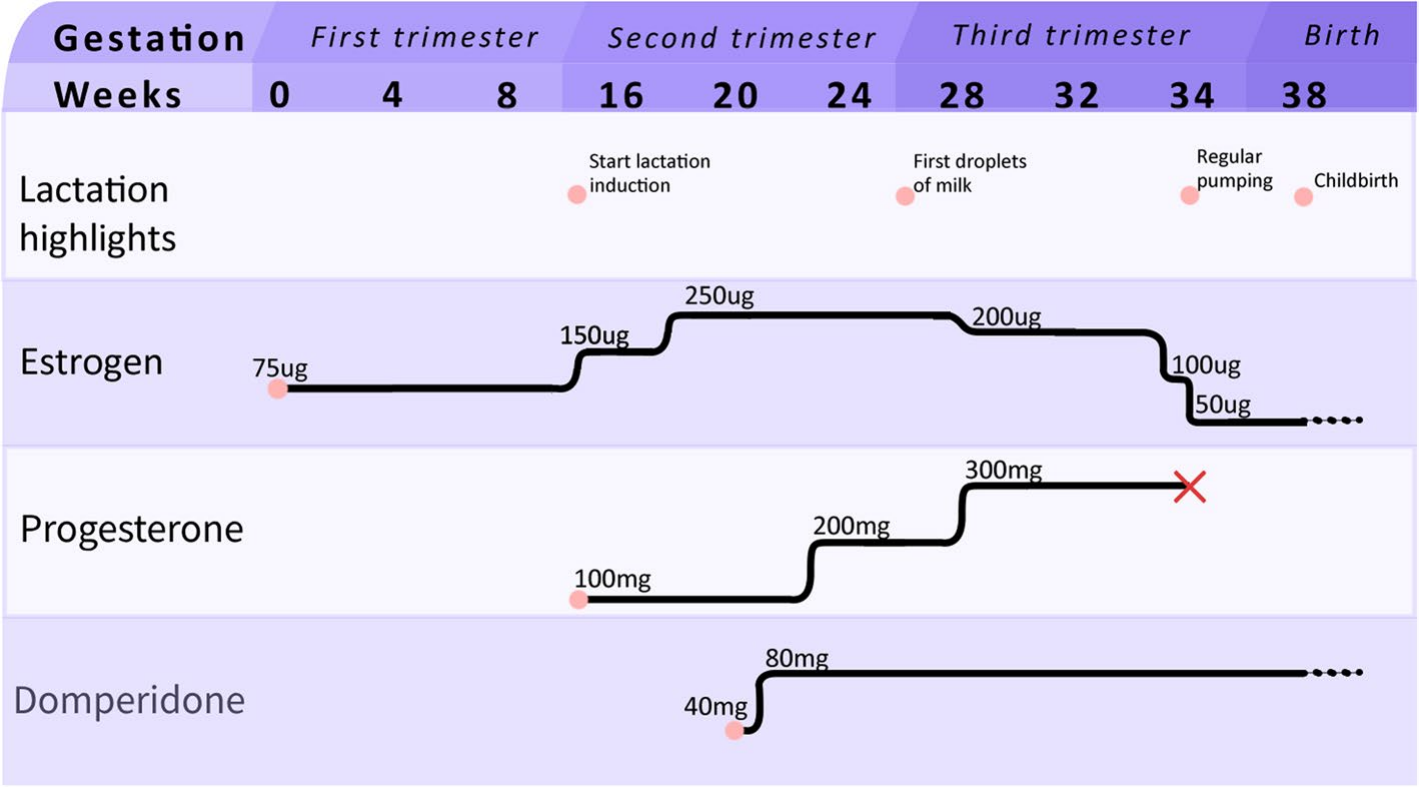
Summary of highlights in treatment regime

Our patient reported that lactation induction did not have a significant effect on her gender identity, neither did it alter the amount of gender dysphoria experienced. The short duration of lactation and low milk production were not experienced negatively, but she experienced the decision to stop as “tough”. She was happy to have achieved lactation and for her infant to latch on and be comforted a few times. Though she knew that (exclusive) nursing was probably not possible, she expressed that “it was a pity” that the experience of long-term feeding had not been possible. Figure 1 shows a summary of highlights in the treatment regime of the patient.

## Discussion

### Case discussion

This case endorses that lactation induction protocols are also successful in transgender women. Our study is in line with published case reports, in which lactation induction was successful, but the production of milk was not sufficient for exclusive nursing [4, 5, 11]. Nevertheless, as the success of induced lactation is often attributed to parent-infant bonding, rather than the possibility of exclusive chestfeeding, this experience is valuable [25–27].

The success of non-puerperal lactation induction and the amount of milk produced depends on several factors [28, 29]. Physical stimulation of the breast tissue is an important factor in promoting lactation in non-puerperal breasts. In our case, due to a short lingual frenulum, the infant latched suboptimally, causing suckling problems. This caused our patient to discontinue lactation after two weeks. These suckling problems may have contributed to low milk production and a shorter period of lactation compared to cases described by prior reports [4, 5, 11]. Another contributory factor may be the time our patient used a breast pump to stimulate the breasts in comparison to other studies. Though our patient started manual stimulation and expression almost 20 weeks prior to delivery of her child, she only started using a breast pump three weeks before delivery, because labour was induced at approximately 38 weeks of gestation due to gestational diabetes. The period our patient was able to use a breast pump prior to the birth of her child was short compared to at least six weeks described in other reports and may have contributed to the low milk production [4, 5, 11]. Our participant refrained from visiting the lactation consultant provided by the hospital, but chose to be guided by a lactation consultant outside of our hospital, hence we did not discuss pumping advice.

Prenatal development of mammary tissue is similar in both males and females. In cisgender women breast development starts at puberty under influence of estrogen. Breast tissue composition changes during pregnancy and lactation [30]. In transgender women breast development occurs after initiation of gender affirming hormone therapy; typically combining estrogen with an anti-androgen. The breast of transgender women are indistinguishable from those of cisgender women on radiography [30, 31]. Nevertheless it is suggested that compared to cisgender women, transgender women have smaller breast and may not reach full breast maturity [30]. Successful lactation is independent of breast volume [32]. However, studies suggest that women with mammary hypoplasia and inadequate glandular tissue may be at risk for lactation failure [33–38].

In our case and the case presented by Wamboldt 2021, the breast size and maturation has not been described [5]. The cases presented by Reisman 2018 and Weimer 2023 do respectively describe degree of maturation and size [4, 11]. In Weimer’s report the patient also underwent mammogram with ultrasound for a medical reason, which was notable for extremely dense breast tissue [11]. Nevertheless, the breast tissue of lactating transgender women has not been analyzed yet and have not been uniformly described in previous published cases. Therefore, the effect of breast maturation on the success of induced lactation for transgender women is not known.

In our case report we did not analyze the nutritional value of milk produced by our patient. Only one previous report has assessed the milk produced by their patient. Weimer et al. assessed their participant’s milk and found values of fat, lactose, protein and calorie were comparable and even higher than those in term milk produced by cisgender women. They did not assess micronutrients and bioactive factors [11].

### Recommendations for clinical practice

#### Physiology and methods for lactation induction

Increased levels of estrogen, progesterone and prolactin during pregnancy prepare the breast for lactation. Estrogen and progesterone inhibit lactation, but stimulate breast development, as proliferation of glandular tissue is stimulated by estrogen and the proliferation of ducts and lobules are stimulated by progesterone [39]. After childbirth and delivery of the placenta, the levels of estrogen and progesterone drop drastically, allowing prolactin to trigger lactation [40]. For non-puerperal induction of lactation, one has to mimic the physiological changes of breasts that occur due to pregnancy and childbirth. This may comprise both non-pharmacological and pharmacological methods [28]. Lactation induction is most effective if these methods are used in conjunction with one another.

Pharmacological methods rely on mimicking pregnancy by first increasing the estradiol and progesterone dose to optimize breast development and composition for lactation, as would naturally occur in pregnancy.

Subsequently, a galactogogue can be started to increase prolactin levels to eventually initiate and maintain milk production. Most galactogogues interact with dopamine receptors to increase prolactin levels. Different galactogogues are available and the choice for a galactogogue may be based on its side effects, and maternal and neonatal safety. First choice for a galactogogue may be domperidone or metoclopramide, as the efficacy of both these agents for lactation induction are well reported, and are safe for both the parent initiating lactation induction and infant [28, 29, 41–43]. It should be noted that both drugs may have notable side effects that have to be taken in account. Metoclopramide may cause extrapyramidal side effects such as bradykinesia, tremor and other dystonic reactions [41, 44]. These side effects are seldom seen in domperidone, since domperidone crosses the blood–brain barrier in minimal amounts compared to metoclopramide [45]. Domperidone and metoclopramide may cause QT prolongation [46, 47], however this effect is more often seen in domperidone than in metoclopramide [48]. Due to this effect it may be difficult to obtain domperidone in some countries [11]. A systematic review from 2021 reported no significant difference in maternal side effects between domperidone and metoclopramide [43], nevertheless a large observational study from 2018 did find that women reported 3.6 times more side effects using metoclopramide in comparison to domperidone [44],

As a final step, the estradiol and progesterone dose should be decreased to mimic the effects of delivery. This will revert the inhibition of prolactin, causing a rise in prolactin to initiate or enhance lactation.

Non-pharmacological methods may include using a breast pump to stimulate the breasts, manual stimulation of the breasts and optimal infant suckling. Successful lactation induction by solely applying non-pharmacological methods have been reported in cisgender women, however, often non-pharmacological and pharmacological methods are combined.

#### Considerations and recommendations

We suggest a thorough medical history taking prior to initiation of lactation medication. Medication used for lactation induction may have negative side effects and may be contraindicated based on the patient’s medical history. Even for patients who have previously taken estrogen, risks may be altered due to higher dosages for induced lactation. The most important patient factors to consider include: (family) history of thrombosis and smoking over the age of 35 years, as estrogen increases the risk of thrombosis [49], and cardiac irregularities, as both domperidone and metoclopramide may cause QT prolongation [46, 47].

We recommend conducting an electrocardiogram on patients with a (family) history of cardiac irregularities.

Care providers should explore the expectations of the parent desiring induced lactation, and manage these expectations by explaining that while exclusive nursing is often not achievable, it is not the sole purpose of lactation induction.

Some transgender women who desire to induce lactation may use anti-androgen agents. Spironolactone is often used, and though it crosses the placenta and into human milk, it is considered safe during chestfeeding [50–52]. Though these effects have not been described after birth in chestfeeding, it is important to inform and counsel patients using anti-androgen agents who wish to pursue lactation induction about the potential impact of these medications on the genital development of the newborn [50–53]. Taking into account that using anti-androgen agents may have a beneficial effect on gender dysphoria in transgender women and weighing this against the very small chance of it having impact on genital development.

Transgender women pursuing induced lactation should be referred to a lactation consultant to optimize breast stimulation techniques, develop pumping schedules, support in infant latching and chestfeeding positions and for adequate support.

Though the optimal dosage and route of administration of medication is not yet known, we suggest the following treatment regime:

- Intake appointment in an outpatient clinic, preferably before conception or in first trimester. Analyse risk factors, allergies and blood-hormone levels.
- At end of first trimester (12–13 weeks of gestation), after confirmation of viable intrauterine pregnancy, increase estrogen dosage to 150ug once daily and start progesterone 100 mg once daily. This should preferably be started three to five months prior to the expected due date. Start stimulation of breast tissue by massaging, nipple stimulation. Discuss referral to a lactation consultant.
- At approximately 17–18 weeks of gestation, monitor blood hormone levels, and increase estrogen to 250ug once daily.
- At 20 weeks of gestation, start domperidone 10 mg four times daily after two weeks.
- At 21 weeks of gestation, double domperidone to 20 mg four times daily.
- At 23–24 weeks of gestation monitor blood hormone levels, increase progesterone to 100 mg two times daily. Adjust estrogen dosage according to blood-hormone levels.
- At 27–28 weeks of gestation monitor blood hormone levels, increase progesterone to 100 mg three times daily. Adjust estrogen dosage according to blood hormone levels.
- At least six weeks before the expected due date, start manual breast pumping of breast to at least every three to four hours, at least once nightly.
- Four to six weeks before the expected due date, decrease estrogen dose to 100ug once daily and discontinue progesterone.
- At onset of milk production decrease estrogen dosage to 50ug once daily. When milk volume increases an electric pump may be used, preferably a hospital grade double electric breast pump.
- After birth of the newborn continue pumping, including after feeds. Supplemental feedings with infant formula might be necessary if milk produced is not sufficient. If the gestational parent is also nursing, continue pumping at least every three hours.

#### Suggestions for future research

Scientific literature on lactation induction in transgender women remains scarce. Future research should focus on optimal treatment regime, the development of the infants that were chestfed by transgender women and the milk composition produced by transgender women. Furthermore, there should be a focus on if the extent of breast maturation has an influence on the success of induced lactation, as it would be beneficial to adequately counsel transgender women seeking help in lactation induction.

## Conclusions

Chestfeeding has outgrown the traditional image of maternity in cisgender women, and is now available for different family structures. Transgender women may desire to chestfeed their infants.

This case report underlined that lactation induction protocols commonly used for cisgender women are also effective in transgender women. However, the amount of milk produced may not be sufficient for exclusive nursing. Nevertheless, success of induced lactation may be attributed to parent-infant bonding, rather than the possibility of exclusive nursing. Moreover, it may even be beneficial to reduce gender dysphoria in transgender women. Lactation can be induced by applying a combination of non-pharmacological and pharmacological techniques. In the choice for pharmacological agents the side-effects and contraindications should be taken into account.

## Data Availability

The data generated during the current study are not publicly available since this data may be traced back to study participant and anonymity cannot be guaranteed. The data are available from the corresponding author on reasonable request, in which case the data will be adjusted to ensure study participant anonymity.

## Abbreviations

AMAB: Assigned male gender at birth
ICSI: Intracytoplasmic sperm injection

## References

[1] Imaz E (2017). Same-sex parenting, assisted reproduction and gender asymmetry: reflecting on the differential effects of legislation on gay and lesbian family formation in Spain. Reprod Biomed Soc Online.

[2] MacDonald TK (2019). Lactation care for transgender and non-binary patients: Empowering clients and avoiding aversives. J Hum Lact.

[3] Paynter MJ (2019). Medication and facilitation of transgender women’s lactation. J Hum Lact.

[4] Reisman T, Goldstein Z (2018). Case report: induced lactation in a transgender woman. Transgender Health.

[5] Wamboldt R, Shuster S, Sidhu BS (2021). Lactation induction in a transgender woman wanting to breastfeed: case report. J Clin Endocrinol Metab.

[6] Szucs KA, Axline SE, Rosenman MB (2010). Induced lactation and exclusive breast milk feeding of adopted premature twins. J Hum Lact.

[7] Flores-Antón B, García-Lara NR, Pallás-Alonso CR (2017). An adoptive mother who became a human milk donor. J Hum Lact.

[8] Bryant CA (2006). Nursing the adopted infant. J Am Board Fam Med.

[9] Biervliet F, Maguiness S, Hay D, Killick S, Atkin S (2001). Induction of lactation in the intended mother of a surrogate pregnancy: case report. Hum Reprod.

[10] Farhadi R, Philip RK (2017). Induction of lactation in the biological mother after gestational surrogacy of twins: A novel approach and review of literature. Breastfeed Med.

[11] Weimer AK (2023). Lactation induction in a transgender woman: macronutrient analysis and patient perspectives. J Hum Lact.

[12] The Lactation Network - Breastfeeding FAQ for trans and non-binary parents [Available from: https://lactationnetwork.com/blog/breastfeeding-faq-for-trans-and-non-binary-parents/.

[13] Burns. Trans women can breastfeed — here's how 2018 [Available from: https://www.them.us/story/trans-women-breastfeed.

[14] Quora thread - Can a transgender woman breastfeed a baby? [Available from: https://www.quora.com/Can-a-transgender-woman-breastfeed-a-baby.

[15] Trautner E, McCool-Myers M, Joyner AB (2020). Knowledge and practice of induction of lactation in trans women among professionals working in trans health. Int Breastfeed J.

[16] Goldman AS (2007). The immune system in human milk and the developing infant. Breastfeed Med.

[17] Hanson LA, Ahlstedt S, Andersson B, Carlsson B, Fällström SP, Mellander L (1985). Protective factors in milk and the development of the immune system. Pediatrics.

[18] Yolken RH, Peterson JA, Vonderfecht SL, Fouts ET, Midthun K, Newburg D (1992). Human milk mucin inhibits rotavirus replication and prevents experimental gastroenteritis. J Clin Investig.

[19] Ip S, Chung M, Raman G, Chew P, Magula N, DeVine D (2007). Breastfeeding and maternal and infant health outcomes in developed countries. Evid Rep Technol Assess.

[20] Bachrach VRG, Schwarz E, Bachrach LR (2003). Breastfeeding and the risk of hospitalization for respiratory disease in infancy: a meta-analysis. Arch Pediatr Adolesc Med.

[21] Arenz S, Rückerl R, Koletzko B, von Kries R (2004). Breast-feeding and childhood obesity—a systematic review. Int J Obes.

[22] Gribble KD (2006). Mental health, attachment and breastfeeding: implications for adopted children and their mothers. Int Breastfeed J.

[23] Linde K, Lehnig F, Nagl M, Kersting A (2020). The association between breastfeeding and attachment: A systematic review. Midwifery.

[24] Zingler E, Amato AA, Zanatta A, Vogt MdFB, Wanderley MdS, Mariani C, Zaconeta AM. Lactation induction in a commissioned mother by surrogacy: effects on prolactin levels, milk secretion and mother satisfaction. Rev Bras Ginecol Obstetr. 2017;39:86–9. 10.1055/s-0037-1598641 PMC10309413 28257588

[25] Cheales-Siebenaler NJ (1999). Induced lactation in an adoptive mother. J Hum Lact.

[26] Thearle MJ, Weissenberger R (1984). Induced lactation in adoptive mothers. Aust N Z J Obstet Gynaecol.

[27] Auerbach KG (1981). Induced lactation: a study of adoptive nursing by 240 women. Am J Dis Child.

[28] Cazorla-Ortiz G, Obregón-Guitérrez N, Rozas-Garcia MR, Goberna-Tricas J (2020). Methods and success factors of induced lactation: A scoping review. J Hum Lact.

[29] Wittig SL, Spatz DL (2008). Induced lactation: gaining a better understanding. MCN. AmJ Matern Child Nurs..

[30] Reisman T, Goldstein Z, Safer JD (2019). A review of breast development in cisgender women and implications for transgender women. Endocr Pract.

[31] Sonnenblick EB, Shah AD, Goldstein Z, Reisman T (2018). Breast imaging of transgender individuals: a review. Curr Radiol Rep.

[32] Żelaźniewicz A, Pawłowski B (2019). Maternal breast volume in pregnancy and lactation capacity. Am J Phys Anthropol.

[33] Arbour MW, Kessler JL (2013). Mammary hypoplasia: not every breast can produce sufficient milk. J Midwifery Womens Health.

[34] Betzold CM, Hoover KL, Snyder CL (2004). Delayed lactogenesis II: a comparison of four cases. J Midwifery Womens Health.

[35] Bodley V, Powers D (1999). Patient with insufficient glandular tissue experiences milk supply increase attributed to progesterone treatment for luteal phase defect. J Hum Lact.

[36] Duran MS, Spatz DL (2011). A mother with glandular hypoplasia and a late preterm infant. J Hum Lact.

[37] Thorley V. Breast hypoplasia and breastfeeding: a case history. Breastfeeding Review. 2005;13(2):13–16. 16127825

[38] Neifert MR, Seacat JM, Jobe WE (1985). Lactation failure due to insufficient glandular development of the breast. Pediatrics.

[39] Conneely O, Mulac-Jericevic B, Arnett-Mansfield R. Progesterone signaling in mammary gland development. In: Conneely O, Otto C, editors. Progestins and the mammary gland. Berlin: Springer; 2008. p. 175–85.

[40] Czank C, Henderson J, Kent J, Lai C, Hartmann P. Hormonal control of the lactation cycle. In Textbook of human lactation. Hale Publishing; 2007. p. 89–111.

[41] Gabay MP (2002). Galactogogues: medications that induce lactation. J Hum Lact.

[42] Ingram J, Taylor H, Churchill C, Pike A, Greenwood R (2012). Metoclopramide or domperidone for increasing maternal breast milk output: a randomised controlled trial. Arch Dis Child Fetal Neonatal Ed.

[43] Shen Q, Khan KS, Du M-C, Du W-W, Ouyang Y-Q (2021). Efficacy and safety of domperidone and metoclopramide in breastfeeding: A systematic review and meta-analysis. Breastfeed Med.

[44] Hale TW, Kendall-Tackett K, Cong Z (2018). Domperidone versus metoclopramide: self-reported side effects in a large sample of breastfeeding mothers who used these medications to increase milk production. Clinical Lactation.

[45] Breuil L, Goutal S, Marie S, Del Vecchio A, Audisio D, Soyer A, et al. Comparison of the blood-brain barrier transport and vulnerability to p-glycoprotein-mediated drug-drug interaction of domperidone versus metoclopramide assessed using in vitro assay and PET imaging. Pharmaceutics. 2022;14(8):1658. 10.3390/pharmaceutics14081658 PMC9412994 36015284

[46] Elli̇dokuz E, Kaya D. The effect of metoclopramide on QT dynamicity: double‐blind, placebo‐controlled, cross‐over study in healthy male volunteers. Alimentary Pharmacology & Therapeutics. 2003;18(1):151–5. 10.1046/j.1365-2036.2003.01641.x 12848637

[47] Smolina K, Mintzes B, Hanley GE, Oberlander TF, Morgan SG (2016). The association between domperidone and ventricular arrhythmia in the postpartum period. Pharmacoepidemiol Drug Saf.

[48] Claassen S, Zünkler BJ (2005). Comparison of the effects of metoclopramide and domperidone on HERG channels. Pharmacology.

[49] Connelly PJ, Marie Freel E, Perry C, Ewan J, Touyz RM, Currie G, Delles C (2019). Gender-affirming hormone therapy, vascular health and cardiovascular disease in transgender adults. Hypertension.

[50] Al-Hadiya BM, Belal F, Asiri YA, Gubara OA. Spironolactone. In: Analytical profiles of drug substances and excipients. Academic Press; 2002. p. 261-320.

[51] Bijwerkingen centrum lareb. Kaliumsparende diuretica (plastabletten) tijdens de borstvoedingsperiode [Potassium-sparing diuretics during breastfeeding] 2019 [Available from: https://www.lareb.nl/mvm-kennis-pagina?id=241&pagina=1&zoekterm=spironolacton&zwangerschap=true&borstvoeding=true&naam=Kaliumsparende%20diuretica%20(plastabletten)%20tijdens%20de%20borstvoedingsperiode.

[52] American Academy of Pediatrics Committee on Drugs (2001). Transfer of drugs and other chemicals into human milk. Pediatrics.

[53] Shah A, editor Ambiguous genitalia in a newborn with spironolactone exposure. 93rd Annual Meeting of the Endocrine Society Boston; 2011.

